# Characterization of pancreatic lesions from MT-*tgf*α, Ela-*myc *and MT-*tgf*α/Ela-*myc *single and double transgenic mice

**DOI:** 10.1186/1477-3163-5-19

**Published:** 2006-07-05

**Authors:** Dezhong Joshua Liao, Yong Wang, Jiusheng Wu, Nazmi Volkan Adsay, David Grignon, Fayyaz Khanani, Fazlul H Sarkar

**Affiliations:** 1 Department of Pathology, Wayne State University School of Medicine, And Karmanos Cancer Institute, 110 E. Warren Avenue, Detroit, MI 48201

## Abstract

In order to identify good animal models for investigating therapeutic and preventive strategies for pancreatic cancer, we analyzed pancreatic lesions from several transgenic models and made a series of novel findings. Female MT-*tgf*α mice of the MT100 line developed pancreatic proliferation, acinar-ductal metaplasia, multilocular cystic neoplasms, ductal adenocarcinomas and prominent fibrosis, while the lesions in males were less severe. MT-*tgf*α-ES transgenic lines of both sexes developed slowly progressing lesions that were similar to what was seen in MT100 males. In both MT100 and MT-*tgf*α-ES lines, TGFα transgene was expressed mainly in proliferating ductal cells. Ela-*myc *transgenic mice with a mixed C57BL/6, SJL and FVB genetic background developed pancreatic tumors at 2–7 months of age, and half of the tumors were ductal adenocarcinomas, similar to what was reported originally by Sandgren *et al *[1]. However, in 20% of the mice, the tumors metastasized to the liver. MT100/Ela-*myc *and MT-*tgf*α-ES/Ela-*myc *double transgenic mice developed not only acinar carcinomas and mixed carcinomas as previously reported but also various ductal-originated lesions, including multilocular cystic neoplasms and ductal adenocarcinomas. The double transgenic tumors were more malignant and metastasized to the liver at a higher frequency (33%) compared with the Ela-*myc *tumors. Sequencing of the coding region of *p16ink4*, k-*ras *and *Rb *cDNA in small numbers of pancreatic tumors did not identify mutations. The short latency for tumor development, the variety of tumor morphology and the liver metastases seen in Ela-*myc *and MT-*tgf*α/Ela-*myc *mice make these animals good models for investigating new therapeutic and preventive strategies for pancreatic cancer.

## Background

Pancreatic cancer is the fourth leading cause of cancer death in the United States and many other western countries [2,3]. The five-year survival rate is less than 5% in most countries [4]. About 75% of human pancreatic cancer is ductal adenocarcinomas, whereas acinar cell carcinomas and other histological types are less common [5]. An important morphologic feature is that pancreatic ductal adenocarcinomas are frequently associated with prominent fibrosis and multiple cysts [4,6,7]. The cell origin of ductal adenocarcinomas is still under debate [8]. Studies with several experimental animal models suggest that it may derive from metaplasia (transdifferentiation) of acinar cells or even islet endocrine cells [9,10]. In human cases, however, hyperplastic and dysplastic epithelial lesions of the pancreatic ducts have been observed frequently in association with ductal adenocarcinomas [5]. Several studies have suggested a strong association of severely dysplastic ductal lesions with invasive carcinomas [11-14]. More convincingly, a series of pancreatic intraepithelial neoplasia (PanINs) have been developed at a think tank sponsored by National Cancer Institute of the United States as precursors to invasive pancreatic cancer [15]. These lines of evidence suggest that ductal cells may be the origin of ductal adenocarcinomas.

Concomitant expression of epidermal growth factor receptor (EGFR) with its ligand, such as EGF, transforming growth factor α (TGFα) or amphiregulin has been associated with decreased patient survival in pancreatic cancer [16]. In one report, strong TGFα immunoreactivity was found in 95% of pancreatic tumors, whereas EGF immunoreactivity was observed in only 12% of the tumors [17]. Similarly, carcinogen-induced pancreatic cancer in the hamster and rat expressed only TGFα and EGFR, but not EGF [18,19]. Data from these human and animal studies are in line with a generally accepted notion that TGFα is the preferred trophic factor over other EGFR ligands for normal ductal cells and cancer cells in the pancreas [20-23]. Several lines of transgenic mice have been established to study the effects of TGFα on pancreatic carcinogenesis, of which the *tgf*α transgenic mouse using elastase-1 gene promoter (Ela-*tgf*α) has been studied extensively [10,24]. The pancreas of these Ela-*tgf*α mice develops not only pronounced fibrosis but also obvious ductal metaplasia of acinar cells because the Ela-promoter targets the transgene mainly to the acinar cells [24,25]. These acinar-derived ductal cells show progressive proliferation and dysplasia. At one year of age or older, about 25–30% of the animals develop pancreatic tumors, but the majority of them are acinar cell carcinomas. Several other *tgf*α transgenic mouse lines were established using metallothionin-1 gene promoter (MT-*tgf*α) [26], but these mice were much less studied for pancreatic lesions, although they were frequently used for studies of carcinogenesis of the mammary gland [27,28] and liver [29].

Overexpression of c-*myc *mRNA [30] and protein [31] has been found in about 50% and 43.5% of human pancreatic ductal adenocarcinomas, respectively, with about 32.3% of the samples bearing c-*myc *gene amplification [31]. Another genetic analysis of 31 human pancreatic cancer cell lines also showed that 54% of the cell lines analyzed had c-*myc *gene amplification [32]. These data suggest that overexpression or amplification of c-*myc *may play an important role in the development or progression of human pancreatic cancer [33], although there are still relatively few immunohistochemical data to verify whether c-Myc protein levels are also increased correspondingly. Animal studies have revealed that pancreastic cancer induced by chemical carcinogens in the rat also manifests increased c-*myc *expression [34,35]. A more direct and convincing evidence for a critical role of c-*myc *in pancreatic carcinogenesis comes from transgenic mice. Mice carrying c-*myc *transgene under Ela-promoter develop pancreatic cancer with 100% penetrance at an early (2–7 months) age [1]. One-half of the pancreatic tumors are acinar cell carcinomas, while the remaining one-half are ductal adenocarcinomas or mixed ductal and acinar carcinomas [1]. Although this Ela-*myc *mouse is the first, and seemingly the only, single-transgene model that gives rise to frank pancreatic tumors with ductal elements in the shortest latency period compared with other single-transgene models [1,36], the pathological characterization of the pancreatic lesions from this model has not yet been described in detail.

Besides the Ela-*myc *mice, several other transgenic mouse models have also been generated for study of exocrine pancreatic cancers [37-42]. However, because currently no gene promoter/enhancer has been known to be specific for pancreatic ductal cell [40], all these transgenic mouse models share a common deficiency: If the transgene is specifically targeted to the pancreas, such as when driven by Ela-promoter, it is dominantly expressed in the acinar cells. Conversely, if the transgene is driven by a promoter specific for ductal cells, its expression is not pancreas specific and is usually at low levels. For instance, the MT-promoter targets the transgene to the mammary gland, liver and pancreas while cytokeratin 19 gene promoter targets the transgene to the stomach, pancreas and probably other organs as well [43]; both promoters are much weaker than the Ela-promoter. Most of the currently existing transgenic mouse models of pancreatic carcinogenesis produce only acinar-ductal metaplasia and ductal proliferation, with zero or very low penetrance of developing frank pancreatic tumors, unless the transgenic mice are concomitantly deficient in certain tumor suppressor genes such as *Ink4/Arf *or p*53 *[43-49]. For instance, mice with both Cre-mediated k-*ras *mutation and *Ink4a/Arf *gene deficiency develop metastatic pancreatic ductal adenocarcinomas [44]. This animal model requires that a mouse concomitantly bears at least four transgene alleles, i.e. *Pdx1-Cre*, *LSL-Kras*^*G12D *^and homozygous *Ink4a/Arf*^*lox*/*lox*^, and thus involves extensive animal breeding and genotyping. Crossing two heterozygous breeders (e.g. Ela-*myc *male × Ela-*myc *female) may increase the frequency of pups that are transgene carriers. However, pups bred by this procedure may not be used for testing new therapeutic or preventive methods, because it will produce a mixture of homozygous and heterozygous pups. It is technically difficult, if not impossible, to distinguish homozygosites from heterozygotes for a large number of pups, and homozygous carriers of transgene may show different sensitivity to the tested agents compared with heterozygotes. This concern becomes an issue when the animals are used for testing therapeutic or preventive agents, although it may not be an issue for study of carcinogenic mechanisms.

Before a gene promoter specific for pancreatic ductal cells is identified, the best choice may still be to use mice expressing double oncogenes driven individually by a pancreas-specific promoter such as Ela-promoter and by a ductal cell dominant promoter such as MT-promoter. We used this strategy to study pancreatic carcinogenesis by crossing MT-*tgf*α and Ela-*myc *mice to create MT-*tgf*α/Ela-*myc *mice, considering that the Ela-*myc *transgene might also be expressed in the ductal cells, as reflected by the appearance of various ductal lesions in the Ela-*myc *pancreas, and thus might synergize with the MT-*tgf*α transgene to induce ductal cell carcinogenesis. This report summarizes the novel findings from these single and double transgenic mice.

## Materials and methods

The MT100 line of MT-*tgf*α transgenic mouse with FVB/N genetic background [26] was originally purchased from Jackson Laboratories and maintained at our laboratory in FVB/N background. In addition, we also received one male MT-*tgf*α transgenic mouse [50] and one male Ela-*myc *transgenic mouse [1], all in C57BL/6 × SJL background, from Dr. Eric Sandgren at University of Wisconsin-Madison. In this study the MT-*tgf*α mouse from Dr. Eric Sandgren is defined with the initial of his name as MT-*tgf*α-ES line, in order to distinguish it from the MT100 line. The FVB mice used for breeding were purchased from Jackson Laboratories. The breeding procedure for each single or double transgenic line is described accordingly in the result section.

Paraffin blocks of 15 cases of human pancreatic ductal adenocarcinomas were retrieved from the Pathology Tissue Repository of Harper University Hospital at Wayne State University, under a protocol approved by the human investigate committee of the University. Criteria for case selection were histologically proved ductal adenocarcinomas and no major treatment before surgical removal of the tumor. Serial sections in 5 μm thickness were prepared from each tissue block and were immunohistochemically stained for TGFα or c-Myc. Sections from mouse pancreatic tissue were prepared in the same way. The primary TGFα antibody was Ab-2 from Oncogene Research Products, San Diego, CA and was used at 1:150 dilution. The c-Myc antibodies were 9E10 monoclonal (from Sigma, St Louis, MO) used at 1:150 dilution and C19 polyclonal (from Santa Cruz Biotech. Inc, Santa Cruz, CA) used at 1:80 dilution. An Avidin-biotin-complex (ABC) method was used for the staining, as described previously [51,52]. A normal rabbit IgG and a normal mouse IgG were used to replace the primary antibodies in the mock staining.

Mouse pancreatic tumor tissues that were kept frozen at -80°C were used for extraction of total cellular RNAs using the RNeasy kit from Qiagen (Valencia, CA). The extracted total RNAs were immediately converted into cDNAs using the TaqMan Reverse Transcription kit from Applied Biosystems (Branchburg, NJ). The cDNAs were then used as PCR templates for the amplification of *p16ink4a*, k-*ras *and *Rb*. For *p16ink4a*, the primer pair is p16-L2 (TCACAGTGAGGCCGCCGCTGAG)/p16-R592 (AGCTCTGCTCTTGGGATTGG) that covers the whole coding sequence. For *k-ras*, the primer pair is KRAS-L140 (TGAGGCGCGGCGGCTCCG)/KRAS-R878 (CTGACAGTTTGCACGAACAGAAG) that also spans the whole coding sequence. For Rb1, the coding sequence was amplified as three fragments using the following overlapping primer pairs: Rb-L57 (CGCGCCTCCCTCGGCTGCT)/Rb-R1200 (GAGTGTGTGGAGTAACCACG), Rb-L926 (GTGTAATATAGATGAGGTGAA)/Rb-R2071 (AGTGTATTTAGTCGGAGATAT), and Rb-L2011 (CCTCCCTTGCCCTGTTTTAC)/Rb-R2919 (CCATGAGCCAGGAGTCTGGT). The amplified PCR products were subsequently subjected to DNA sequencing analysis by the Sequencing Core Facilities of Wayne State University. When DNA sequencing revealed nucleotide alterations that lead to amino acid changes, the above whole procedure was repeated starting from amplifying PCR fragments using the cDNAs. This would rule out the possibility that the PCR process introduced the alterations randomly.

## Results

### Features of pancreatic lesions in MT-*tgf*α transgenic mice

During our original study of mammary gland tumorigenesis involving the MT100 line of MT-*tgf*α transgenic mice (FVB/N background), we accidentally found that the female mice had a much shorter life span than their male littermates. While males survived well and were still very healthy at 14 months of age (older mice were not monitored systematically), most females died during 6–8 months of age, showing progressively decreased activity about one month before death. Because the original report of this transgenic line described pathologic alterations in the liver and pancreas as well [26], we performed autopsies of the dead animals and also sacrificed some transgenic females (in total of 20 animals) at the time when they were less active or moribund (at the age of 6–8 months). We also sacrificed another 20 transgenic females at 2–3 months of age as controls of young age and 8 animals at different time points during 3–5 months of age. Female wild type littermates at 2–3 months (5 animals) and 7 months (4 animals) of age were also included as normal controls. The liver and pancreas were examined macroscopically and histologically.

**Figure 1:**
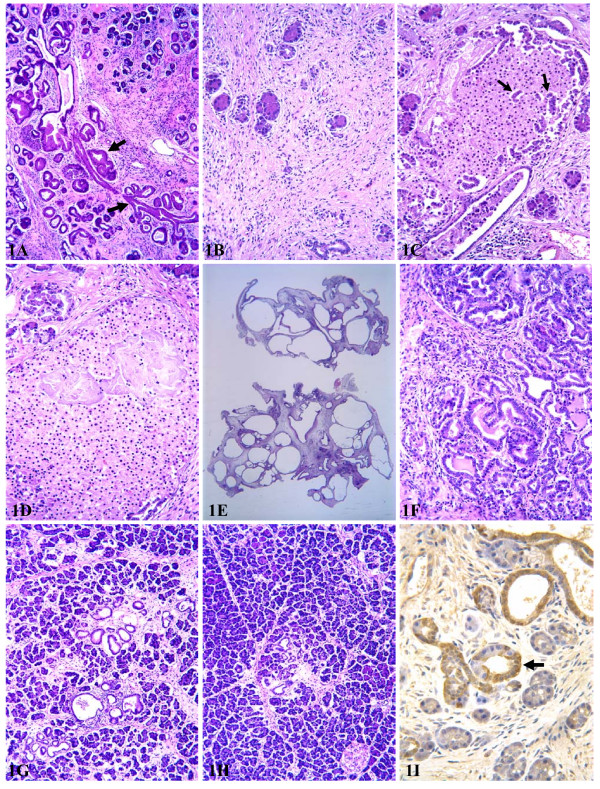
Histological alterations of the pancreas from MT-*tgf*α transgenic mice. **A**: a representative area of the pancreas from a 3-month-old female MT100 mouse, showing formation of fibrosis, proliferating ductal lesions (arrow) that manifest intraductal mucinous changes. **B**: a pancreas from an 8-month-old female MT100 mouse showing prominent fibrosis and severe loss of acini, with features of chronic pancreatitis. **C**: hyperplastic ducts (arrows) in an islet resembling ductuloinsular body in humans, from a 6-month-old female MT100 mouse. **D**: Large necrotic areas seen in an islet, from a 6-month-old female MT100 mouse. **E**: Low magnification of multiocular cystic neoplasms from an 8-month-old female MT100 mouse. **F**: Well differentiated ductal adenocarcinomas with chronic pancreatitis, from a 6-month-old female MT100 mouse. **G**: A representative area of the pancreas from a 9-month-old female MT-*tgf*α-ES mouse, showing early formation of small cysts and fibrosis. **H**: A representative area of the pancreas from a 9-month-old male MT-*tgf*α-ES mouse, which was a littermate of the female shown in G. **I**: Immunohistochemical staining of TGFα in a 3-month-old female MT100 mouse, noting that within the same acinar-ductal loop, only the ductal cells, but not the acinar cells, are positive.

The pancreas of female MT100 mice at 2–3 months appeared as a solid organ whereas the pancreas of the wild type littermates appeared as loose tissue. Histological observation showed progressive death of acinar cells, while ductal cells proliferated progressively to form lesions that resembled mouse pancreatic intraductal neoplasia (mouse PanINs) described in the literature [43,45,46], but these lesions are collectively coined as "ductal proliferation" herein (fig 1A), since it is currently unclear whether such lesions in the mouse are also cancer precursors as PanINs in humans. Progressive fibrosis was associated with the continuing acinar cell death and ductal proliferation (fig 1A). Acinar-ductal metaplasia was observed, although it seemed to be much less evident than what was described for Ela-*tgf*α mice [10,24,25].

In 15 of the 20 MT100 females at age of 6–8 months, the pancreas became much smaller, roughly about 10% to 20% of the size of the pancreas at age of 2–3 months. At age of 6–8 months, the pancreas lost about 90% of its acini and basically consisted of only fibrous tissue and proliferating ducts, with features of chronic pancreatitis (fig 1B). Thus, the severe loss of acini is likely the cause of death, and the female mice might be a good animal model of chronic pancreatitis. Interestingly, proliferating ducts also appeared in islets (arrow in fig 1C), resembling the ductuloinsular body in humans. This trait indicates that in this transgenic line, either the islets might contain certain stem cells with multiple potential that could proliferate to form ductal lesions or some endocrine cells might be capable of undergoing ductal metaplasia and forming ductal lesions, as suggested by certain animal models [8,9]. Areas of cell death (likely necrosis) were also observed frequently in islets (fig 1D). In the other five older females the pancreas showed the opposite changes macroscopically, i.e. enlargement, due to the fact that proliferating ducts formed multiple cysts; some cysts were filled with liquid and developed multilocular cystic neoplasms (fig 1E) that were as large as 1.5 cm^3^. Areas of well differentiated ductal adenocarcinomas (fig 1F) were observed in two of the 20 older mice studied.

We also sacrificed 30 and 20 male MT100 mice at ages of 12–14 months and 2–3 months, respectively, and an additional 15 male transgenic mice in between ages. We also sacrificed five 3-month-old and five 13-month-old male wild type littermates as normal controls. Male MT100 mice at age of 2–3 months started to show death of acinar cells, ductal metaplasia of acinar cells, proliferation of ductal cells, and formation of fibrosis, but these alterations were not as obvious as seen in the age-matched females. Opposite to what was seen in female MT100 mice, the pancreas of males became larger with increased age and body weight. Thus, the pancreas of the males at age of 12–14 months was much larger than the pancreas of males at 2–3 months of age and showed pronounced fibrosis and ductal proliferation and metaplasia, although these lesions were still much less severe compared with the pancreas of females at 6–8 months of age. No multilocular cystic neoplasms and ductal adenocarcinomas were observed in male mice.

We also crossed a male MT-*tgf*α-ES line (C57BL/6 × SJL background) with a female FVB mouse. The F1 transgene carriers were crossed with F1 wild type mice to produce F2 animals and some F2 mice were crossed together to produce F3 mice. Both F1 and F2 MT-*tgf*α-ES carriers did not show obvious sex difference in survival since all 30 animals of both sexes survived well over 14 months of age (older mice were not monitored systematically). In addition, we also sacrificed another 37 F2 and F3 mice (18 males and 19 females) at different time points during 2–10 months of age. Histologically, the pancreas of these mice manifested ductal metaplasia of acinar cells, proliferation of ductal cells, formation of small cysts, and fibrosis, similar to what was reported originally by Sandgren *et al*. for the MT-*tgf*α-ES line with C57BL/6 × SJL genetic background [50]. However, these alterations not only occurred much later (after 4 months of age) but also progressed much slower compared with age- and sex-matched MT100 mice. Female predilection could be discerned after 9 months of age (fig 1G*vs *1H), but even at 14 months of age the sex difference was still not as pronounced as in MT100 mice at age of 6–8 months, because the lesions in female MT-*tgf*α-ES mice were much less severe. No multilocular cystic neoplasm or ductal adenocarcinoma was discerned in MT-*tgf*α-ES mice, but mice older than 14 months were not examined. Unlike what was reported by Sandgren *et al*. [53], we did not observe any macroscopic liver tumor in our MT-*tgf*α-ES mice with mixed C57BL/6, SJL and FVB background.

Immunohistochemistry showed a preferential staining of TGFα in ductal cells in both MT100 and MT-*tgf*α-ES lines (fig. 1I). The staining was strong in most proliferating ductal cells but was weak or undetectable in acinar cells. Even within the same acinar-ductal loop (arrow in fig. 1I), the ductal cells showed intense positive staining while the acinar cells were negative.

### Features of pancreatic lesions from Ela-*myc *transgenic mice

**Figure 2:**
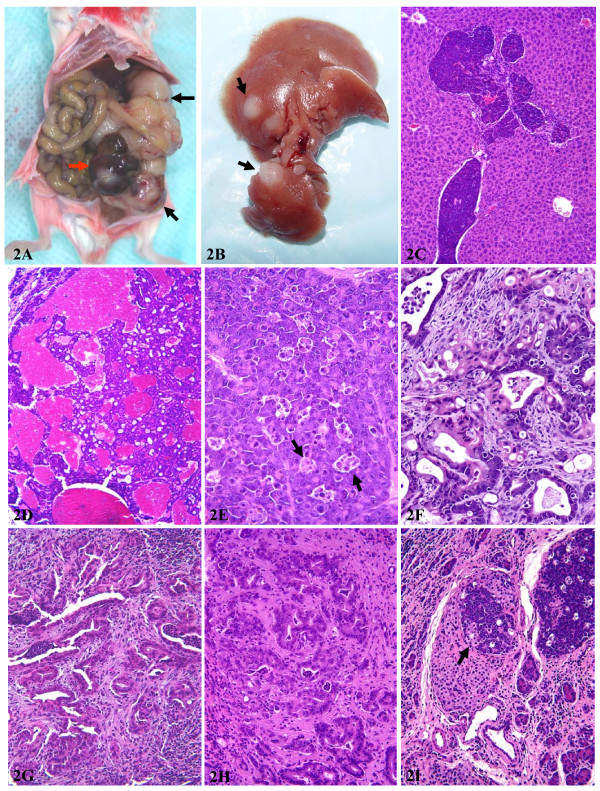
Alterations of the pancreas from Ela-*myc *transgenic mice. **A**: a photo showing a huge nodular pancreatic tumor (arrows). Note that one tumor nodule is in red color while other tumor nodules are in white color. **B**: liver metastases (arrows) of a pancreatic tumor. **C**: histological examination confirming that the liver tumors are pancreatic origin (acinar cell carcinoma). **D**: a typical histology of acinar cell carcinoma that shows red color macroscopically. **E**: a typical histology of the acinar cell carcinoma that shows white color macroscopically. Note that there are many apoptotic cells that are organized in clusters, coined as "death cell islands" (arrows). **F**: a typical area of mixed acinar and ductal adenocarcinomas. **G**: a pancreatic ductal adenocarcinoma. Note that the tumor contains abundant stroma. **H**: another typical ductal adenocarcinoma. **I**: an acinar cell carcinoma within an islet (arrow).

We crossed one male Ela-*myc *mouse (C57BL/6 × SJL background) with a female FVB mouse and then crossed F1 pups together to produce F2 transgene carriers. Some F2 animals were also crossed together to produce F3 mice. Sixty F1, F2 and F3 Ela-*myc *mice of both sexes showed enlarged abdomen, a sign of bearing large pancreatic tumor, at ages between 2–7 months as originally reported by Sandrgen *et al*. [1]. The animals were sacrificed at this period of age when the tumor burden reached the ethical limit or the animals became weak and showed decreased activity. Most tumors weighed 3–4 grams (fig 2A). Peritoneal metastatic tumor seeds were found in 41 (68%) animals. Although Sandgren *et al*. did not mention liver metastasis in their original report of this transgenic line with C57BL/6 × SJL background [1], in 12 (20%) mice we observed macroscopic liver metastasis (fig 2B), which was confirmed histologically to be of pancreatic origin (fig 2C). All the peritoneal and liver metastases occurred at advanced stage, i.e. 4–7 months of age. Microscopic metastasis in the liver was not examined systematically but likely existed in some of the animals that did not show macroscopic metastasis. No macroscopic metastasis in the lung or other organs was found.

At the time of sacrifice, the tumor from each animal manifested multiple nodules, presumably a reflection of multiple original tumors (fig 2A). Some tumor nodules were fish-meat-like white, a typical sign of solid cancer in humans, while some other tumor nodules were a deep red color due to hemorrhage within the tumor (fig 1A). Histologically, about one-half of the tumors were pure acinar cell carcinomas (fig 2D and 2E), while the other one-half were mixed ductal and acinar carcinomas that could be manifested either as mixed cell carcinomas (fig 2F) or as patches of ductal adenocarcinomas (fig 2G and 2H) and other patches of acinar cell carcinomas, similar to what was described in Sandgren *et al's *original report [1]. While acinar tumors could be either white or red in color, all ductal adenocarcinomas were white in color. Interestingly, some acinar tumors also appeared in endocrine islets (arrow in fig 2I). Acinar cell carcinomas manifested many apoptotic cells (fig 2E) and large areas of necrosis. The apoptotic cells were not randomly distributed but, instead, were usually organized in clusters that were coined "dead cell islands" in the description of apoptotic cells in the mammary tumors of MMTV-c-*myc *transgenic mice [28]. Acinar cell tumors contained little stroma (fig 2E), but invasive growth into the adjacent stroma was observed at advanced stages. Unlike acinar cell tumors, ductal tumor cells were usually disseminated in the dense stromal tissue, (fig 2F and 2G), similar to desmoplasia observed in human pancreatic ductal adenocarcinomas. Some ductal areas had some acidophilic cells reminiscent of oncocytic changes. Apoptotic cells and large necrotic areas were much less frequently observed in ductal tumors, compared with acinar tumors.

We also sacrificed 10 mice at 2 months of age that had not yet shown enlarged abdomen and found that 4 of the animals already had small cancer nodules (1–6 mm in diameter). Two of these 4 mice had only one tumor nodule in the head of the pancreas while the other two mice had two tumor nodules, one at the head and the other at the tail of the pancreas. It is likely that the head of pancreas is more susceptible for the tumor development, although the c-*myc *transgene can also induce tumors in other parts of the pancreas. This phenomenon was not mentioned in the original report by Sandgren *et al *[1] and thus whether it also occurred in the original Ela-*myc *mice with C57BL/6 × SJL background is unclear.

### Features of pancreatic lesions in MT-*tgf*α/Ela-*myc *double transgenic mice

We crossed Ela-*myc *mice with MT100 mice bred during the above-described procedure and obtained 21 double transgenic animals with mixed C57BL/6, SJL and FVB background. The majority (16; 76%) of these mice were sacrificed at 3–5 months of age, but the others were sacrificed as early as 2 months or as late as 6 months of age, because they became weak or the tumor burden reached the ethical limit. It seemed that the double transgenic tumors developed earlier or grew faster than the Ela-myc tumors, but the difference was not statistically significant in this small number of tumors.

**Figure 3:**
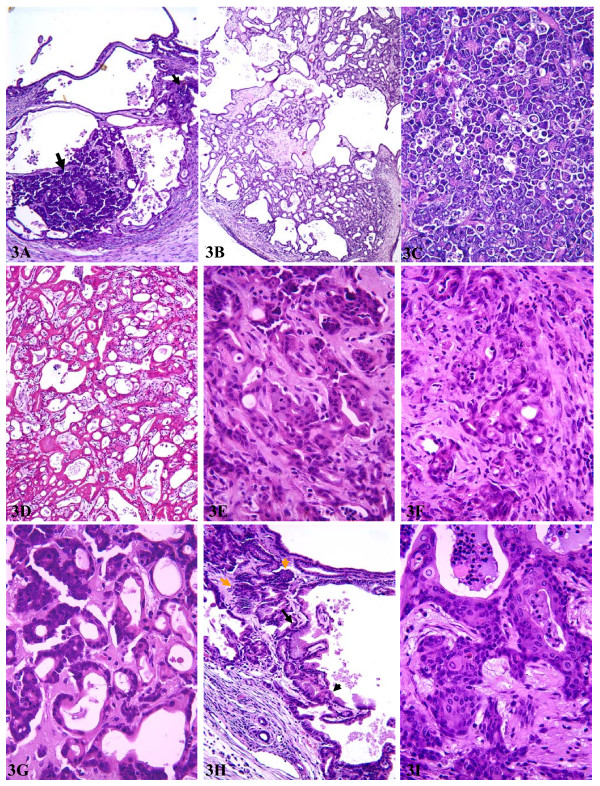
Histological alterations of the pancreas from MT-*tgf*α/Ela-*myc *double transgenic mice. **A**: multilocular cystic neoplasms mixed with acinar tumor cells (arrows). **B**: a large benign multilocular cystic neoplasm. **C**: a typical acinar cell carcinoma. **D**: one type of ductal adenocarcinoma. **E**: another type of ductal adenocarinoma. **F**: a much less differentiated ductal adenocarcinoma with feature of desmoplasia. **G**: a mixed acinar and ductal adenocarcinoma. **H**: an area showing feature of mouse PanIN3 or ductal adenocarcinomas (dark arrows) with acinar tumor cells (yellow arrows) in the surrounding. **I**: a tumor area showing squamous differentiation.

Macroscopically, the tumors looked similar to those from Ela-*myc *mice. However, usually part of the white color tumors, but not the red color ones, manifested cystic features. Although Sandgen *et al*. did not observe ductal elements in their Ela-*tgf*α/Ela-*myc *and MT-100/Ela-*myc *double transgenic mice [53], we found that the pancreas of our double transgenic mice manifested a combination of lesions seen individually in the MT-*tgf*α mice and the Ela-*myc *mice, including proliferating ducts (fig 3A), multilocular cystic neoplasms (fig 3A and 3B), acinar cell carcinomas (fig 3C), various ductal adenocarcinomas (fig 3D, 3E and 3F) and mixed acinar and ductal carcinomas (fig 3G). Dysplastic ductal lesions were also observed and usually mixed with acinar tumor cells (fig 3H). The ductal lesions, such as multilocular cystic neoplasms and ductal adenocarcinomas, seemed to be more prominent in the female mice than in males. Some tumor cells manifested certain squamous differentiation (fig 3I). Fibrosis was also observed (fig 3B) but was much less severe compared with age- and sex-matched mice of MT100 line. Similar to what was seen in Ela-*myc *mice, one-half of the tumors were acinar cell carcinomas and another one-half were ductal tumors or mixed ductal and acinar cell tumors. In general, double transgenic tumors of either acinar or ductal cell origin were more malignant, i.e. less differentiated, than the tumors from Ela-*myc *mice, as previously reported by Sandrgen *et al*. [53]. Acinar cell tumors contained little stroma, similar to Ela-*myc *acinar tumors, whereas ductal tumors were abundant with stromal tissue, somewhat resembling pancreatic ductal adenocarcinomas in humans. Necrotic areas and apoptotic cells appeared in both acinar tumors and ductal tumors but were more frequent in acinar tumors.

We observed macroscopic liver metastasis in 7 of 21 (33%) double transgenic animals. This rate is significantly (χ^2 ^test, p < 0.05) higher than the metastatic rate (20%) in Ela-*myc *mice. Microscopic metastasis was not systematically examined but likely existed in some of those animals that did not show macroscopic metastasis. Peritoneal tumor seeds were also frequently found, as seen in Ela-*myc *mice. No macroscopic metastasis in the lung or other organs was found.

We also crossed some Ela-*myc *mice with the MT-*tgf*α-ES line and got 9 double transgenic pups. The pancreatic lesions of these mice were in general similar to the MT100/Ela-*myc *mice described above, but less multilocular cystic neoplasms were observed compared with MT100/Ela-*myc *mice. Two of these mice appeared to have macroscopic liver metastasis.

### Expression of c-Myc and TGFα in human ducal pancreatic adenocarcinomas

**Figure 4:**
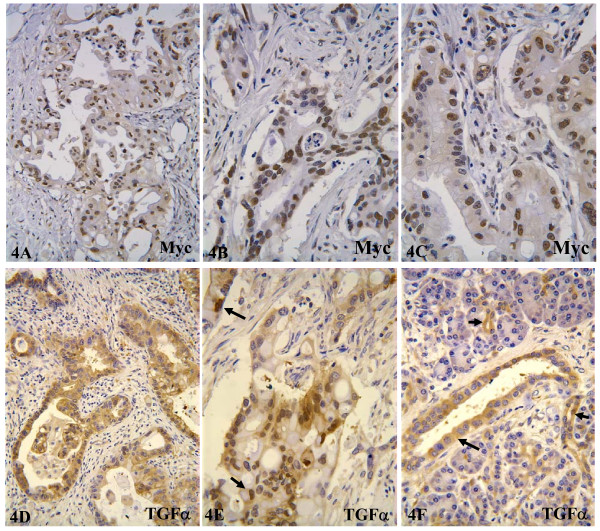
Immunohistochemical staining for c-Myc and TGFα in human pancreatic ductal adenocarcinomas. **4A**, **4B **and **4C**: c-Myc staining showing that most tumor cells manifest positive nuclear staining. **4D **and **4E**: TGFα staining showing most tumor cells are positive for TGFα. Note that the staining is mainly localized in the cytoplasm of most tumor cells, but it is also localized in the nucleus (arrows) of some tumor cells. **4F**: a "normal" area of pancreatic tissue adjacent to cancer, showing that ductal cells (arrows), but not acinar cells, are positive for TGFα.

So far there are still relatively few publications on immunohistochemical data of c-Myc in human pancreatic cancer, and some of the early studies might be limited due to the lack of optimal c-Myc antibodies for immunohistochemistry on paraffin-embedded tissue sections. Therefore, we conducted immunohistochemical staining on a small number of human samples of ductal adenocarcinoma to confirm the relevance of c-Myc oncoprotein to human pancreatic cancer. Immunohistochemical staining was carried out with one monoclonal and one polyclonal antibody. The two antibodies gave rise to very similar staining, and only those tissue areas that showed staining with both antibodies were considered positive. We found that 13 of 15 cases showed moderate to strong staining in about 20–70% of the tumor cells. The staining was mainly localized to the nucleus (fig 4A, 4B and 4C), although many tumor cells also showed weak cytoplasmic staining. Fibroblasts in the stroma were negative. "Normal" pancreatic tissue and pancreatitis tissue adjacent to the tumors were also negative. The mock staining using normal rabbit IgG to replace c-Myc antibodies did no give rise to any staining, confirming the signal specificity of the antibodies.

The staining for TGFα revealed that TGFα was also highly expressed in the 13 cases that were positive for the c-Myc but negative in the 2 cases that failed to show c-Myc staining. It is likely that these two cases might not have been properly fixed or might have had other unknown defects. In these 13 cases, about 30–70% of the tumor cells were moderately to strongly positive for TGFα. In most cancer cells, the staining was localized to the cytoplasm (fig. 4D and 4E), but nuclear staining was also observed in some cancer cells (arrows in fig. 1E), suggesting that TGFα might have transcriptional activity, like EGF [54]. In the adjacent "normal" or pancreatitis tissues that might have certain proliferating potential, many ductal cells, but not acinar cells, were also positive for TGFα (arrows in fig 4F). These results not only dovetail with the thought that TGFα is important for the growth and development of the pancreas but also indicate that the positivity of TGFα may be indicative of ductal cell origin of the cancer. Fibroblasts in the tumor stroma were negative.

### Sequencing of *p16ink4a*, k-*ras *and *Rb *cDNA

Eleven (8 Ela-m*yc *and 3 MT-t*gf*α/Ela-*myc*) tumors were sequenced for the entire coding region of *p16ink4a *cDNA. None of them showed any nucleotide alteration when compared with the NCBI gene bank sequence (accession#: NM_009877). Twelve (9 Ela-*myc *and 3 MT-*tgf*α/Ela-*myc*) tumors were sequenced for the entire coding region of the k-ras cDNA. Eleven of them did not show any nucleotide alterations when compared with the NCBI gene bank sequence (accession#: NM_021284), while one sample had a single nucleotide change (TAC→TAT) at codon 32, which does not change amino acid sequence (fig. 1, panel A) and thus is considered a polymorphism.

Five Ela-*myc *tumors were sequenced for the coding region of the *Rb *gene. All of them showed one nucleotide difference at codons 836 (GAG→GAA), 867 (AAC→AAG) and 868 (GTG→CTG) (panel B and C of fig 1), when compared with the NCBI gene bank sequences (accession# NM_009029 and M26391). Whereas the nucleotide difference at codon 836 did not change the amino acid sequence, the difference at codons 867 and 868 caused changes of two consecutive amino acids, i.e. an asparagine (N) was changed to a lysine (K) at codon 867 and a valine (V) to a leucine (L) at codon 868. Comparison of the Rb protein sequences among mouse, rat and human revealed that the domain that harbors the mouse codons 867 and 868 were highly conserved among these three species. *Rb *cDNA in both rat and human has the lysine (K) and leucine (L) at the positions corresponding to codons 867 and 868 in the mouse *Rb *cDNA, as shown in panel D of Figure 1. Moreover, the mouse genome sequence (access # NC-000080) deposited more recently in the NCBI data base also showed that the mouse *Rb *genomic DNA sequence at these three codons was the same as our sequencing data. We thus considered that the mouse *Rb *cDNA sequences at NCBI gene bank might be wrong. To further clarify this issue, we sequenced the cDNA of a cell line derived from one Ela-*myc *pancreatic tumor (Ela-*myc*-1) that was described in our recent publication [55], as well as the NMuMG cells, a normal mouse mammary epithelial cell line purchased from ATCC. We also sequenced cDNA from a normal mouse kidney tissue. The sequencing data from all these cell lines and kidney tissue showed the same nucleotide sequence as Ela-*myc *tumor tissue. Therefore, it is likely that the *Rb *gene in Ela-m*yc *tumors was not mutated but, instead, the mouse *Rb *cDNA sequences at the NCBI gene bank (accession# NM_009029 and M26391) may have mistakes at these three nucleotides.

## Discussion

Although the MT100 and MT-*tgf*α-ES lines of MT-*tgf*α mice as well as the Ela-*myc *mice have been established for over a decade, so far there are only several published studies using these transgenic mice to address pancreatic carcinogenesis, and detailed characterization of the pancreatic lesions from these mice is still lacking. During a study of mammary carcinogenesis of MT100 mice, we accidentally found, for the first time, the obvious sex differences in the pancreatic lesions of this transgenic line. Bardeesy *et al*. observed multiple cystic lesions with similarly low penetrance in MT42, another MT-*tgf*α transgenic line, but only in the mice that were concomitantly deficient in one allele of *Ink4a *and/or *p53 *gene, not in the MT42 mice with wild type of any of these two genes [56]. The reason for this discrepancy could be due to the facts that their mice were from another transgenic line and had different genetic background, and that the gender of the mice (25 animals) in their study was not described. It is known that in humans various types of cystic neoplasms of the pancreas have malignant potential and show female predominance [57-60]. It deserves further studies whether our observation of female predilection of multilocular cystic neoplasms in the MT100 mice is etiologically or mechanistically relevant to such benign neoplasms in the human pancreas, although the multilocular cystic neoplasms in the mice resemble, histologically, the cystic lesions observed in the human pancreas, as have shown by Bardeesy *et al*. [56]. In our MT100 mice, these well-demarcated multiolcular cystic lesions had loose mesenchyme in the septae, closely resembling ovarian like stroma of human mucinous cystic neoplasm. Moreover, there were luteal type cells, further supporting this hypothesis.

In both MT100 and MT-*tgf*α-ES lines, TGFα is found expressed preferentially in the proliferating ductal lesions, not in the acinar cells or stromal tissue. This expression pattern is different from the dominant expression of the transgenes in the acinar cells of Ela-promoter driven transgenic mice. This difference in the expression patterns may partly explain why the transdifferentiation from acinar cells to ductal cells was not as evident in our MT100 mice as described by Schmid *et al*. for the Ela-*tgf*α mice [10,24,25]. Because Ela-*tgf*α and MT-*tgf*α preferentially target to different cell types, the underlying mechanisms for the formation of fibrosis and the death of acinar cells in these two different transgenic lines may be mechanistically different. It is possible that in MT100 mice, constitutive overexpression of the *tgf*α transgene in the ductal cells causes, perceivably via epithelial-stromal interaction, continuous growth of connective tissue to form fibrosis, which in turn leads to continuous death of acinar cells. It needs to be further explored why and how ductal-derived TGFα induces progressive death of acinar cells and growth of stroma in MT100 mice. On the other hand, MT-*tgf*α/Ela-*myc *double transgenic mice showed much less pronounced fibrosis in the pancreas, compared with MT-*tgf*α mice. An explanation is that overexpressed c-Myc suppresses TGFα induced formation of fibrosis, although TGFα facilitates c-Myc induced pancreatic carcinogenesis by inducing more malignant phenotypes of the tumors. A similarly less evident fibrosis was also observed in the mammary tumors from MT-*tgf*α/MMTV-*myc *double transgenic mice than in the fibrous mammary gland of MT-*tgf*α mice [28]. The molecular mechanism behind this phenomenon is currently unknown.

The finding of liver metastasis of the pancreatic tumors in Ela-*myc *mice is a surprise, since it was not described by Sandgren *et al*. in their original report of this transgenic line [1]. The reason for this different observation is unclear. We intend to consider that the mixed C57BL/6, SJL and FVB genetic background of our animals renders the pancreatic tumor a higher metastatic ability. However, the changed genetic background does not seem to alter the latency for the tumor development, since our animals develop pancreatic tumors at the same age as reported by Sandgren *et al*. [1]. Despite the unexplained discrepancy, this finding makes the Ela-*myc *mouse the first single-transgene model that yields a high metastatic rate. Endogenous expression of mutant k-*ras *in a transgenic model has been shown to cause ductal proliferation that occasionally progresses to frank tumors with invasive and metastatic potential [46]. Another two transgenic mouse lines using Ela-promoter to target k-*ras *mutant or Ela-SV49TAg to the pancreas develop only mouse PanINs [1,43,45,47] but not frank tumors of exocrine origin. Development of frank, metastatic tumors requires combination of expression of k-*ras *mutant or Ela-SV49TAg with the deficiency of the *Ink4a*/*Arf *gene [44,46,47]. The *k-ras mutant*/*Ink4a*^-/- ^model [44] gives rise to metastatic ductal adenocarcinomas and is thus very useful for mechanistic research of pancreatic carcinogenesis. However, it requires that a mouse concomitantly bears four transgene alleles, i.e. *Pdx1-Cre*, *LSL-Kras*^*G12D *^and homozygous *Ink4a/Arf*^*lox*/*lox*^. This model, and another one that involves expression of k-*ras *mutant and p53 knockout, requires extensive animal breeding and genotyping, which greatly limits its use for the purpose of studying new therapeutic or preventive methods or agents. Therefore, several characteristics of Ela-*myc *mouse, i.e. high frequency of ductal adenocarcinomas, high metastatic rate, short latency of carcinogenesis, and the easiness of breeding and genotyping make it a very useful animal model for studying pancreatic carcinogenesis and testing new therapeutic and preventive methods or agents.

Although Sandgren *et al*. did not observe ductal elements in the pancreatic tumors from their Ela-*tgf*α/Ela-*myc *or MT-*tgf*α/Ela-*myc *double transgenic mice [53], we found that proliferating ductal lesions, multilocular cystic neoplasms and ductal adenocarcinomas occurred frequently in the pancreas of our MT-*tgf*α/Ela-*myc *mice. Sandgren *et al*. considered that the reason for the lack of ductal elements could be due to the earlier development of more malignant tumors with reduced life span of these animals [53]. Reduction in the life span seems to be less evident in our double transgenic animals since it does not reach the statistically significant level when compared with the Ela-*myc *mice. In addition, we also found that MT-*tgf*α/Ela-*myc *double transgenic tumors metastasize to the liver at a higher frequency than the Ela-*myc *pancreatic tumors, although the liver metastasis of the double transgenic tumors was, again, not observed in the study of Sandgren *et al*. [53]. Differences in the genetic background of the mice may be one of the explanations for the different observations. This seemingly banal caveat is supported by the facts that Sandgren *et al*. found primary liver tumors in their MT-*tgf*α mice at a high frequency (16 of 27 animals) and in their wild type mice at a low frequency (1 of 20 animals) during 26–104 weeks of age [53], whereas we did not find any primary liver tumors in any of our MT-*tgf*α mice or their wild type littermates up to 12 months of age.

k-*ras*, *Rb *and *p16ink4a *are the genes showing mutations or inactivation at high frequencies in human pancreatic cancer. Surprisingly, although the Ela-*myc *and MT-*tgf*α/Ela-*myc *tumors were highly malignant and have metastatic ability, they did not show mutations in these genes, at least not at high frequencies. Schaeffer et al had also sequenced the k-*ras *in Ela-*myc *tumors and did not find mutation [61].

In summary, this study documents a series of novel findings in several single and double transgenic mouse models established previously by other investigators. Female, but not male, MT-*tgf*α (MT100 line) mice developed multilocular cystic neoplasms and ductal adenocarcinomas at low penetrance. Ela-*myc *mice with mixed genetic background develop pancreatic cancers that metastasize to the liver. MT-*tgf*α/Ela-*myc *dual transgenic mice develop a variety of pancreatic lesions including multilocular cystic neoplasms and ductal adenocarcinomas that are not reported previously. Moreover, the double transgenic tumors metastasize to the liver at a higher frequency than the Ela-*myc *pancreatic tumors. The early formation and the variety of the tumor morphology, as well as the potent metastatic ability seen in the Ela-*myc *and MT-*tgf*α/Ela-*myc *transgenic mice, make them good animal models for testing new therapeutic and preventive regimens or agents for the management of pancreatic cancer.

## Abbreviations

Ela-: elastase-1 gene promoter

MT-: metallothionin-1 gene promoter

MMTV: mouse mammary tumor virus long terminal repeat

PanINs: pancreatic intraductal neoplasia

TGFa: transforming growth factor alpha

## Competing interests

The author(s) declare that they have no competing interests.

## Authors' contributions

DJL is the principle investigator of this study who summarized the data and drafted this manuscript. YW is a postdoctoral fellow who helped with the collection of animal experiment data. JW is a research assistant who performed sequencing of Rb, p16 and k-ras. NVA is a pathologist who helped with characterization of pathologic alterations of the mouse and human pancreatic lesions. DG is a pathologist who helped with characterization of pathologic alterations of the mouse and human pancreatic lesions. FK is a pathologist who helped with characterization of pathologic alterations of the mouse and human pancreatic lesions. FHS is an expert in pancreatic cancer who contributed valuable opinions in the interpretation of the data and help drafting the manuscript. All authors read and approved the final manuscript.

**Figure 5:**
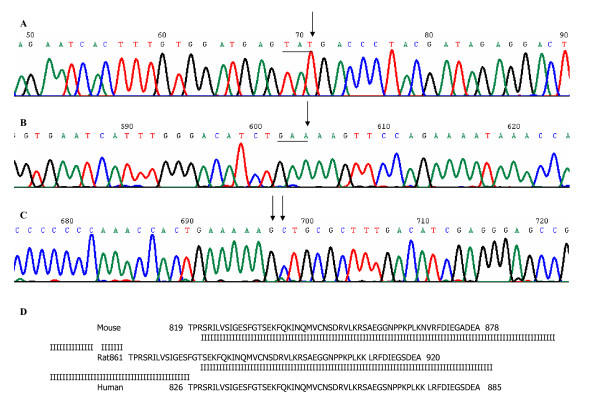
Sequencing data of k-*ras *and *Rb *cDNA. Panel A, chromatogram showing nucleotide change of k-*ras*; panel B and C, chromatograms showing nucleotide changes of *Rb*; panel D, Comparison of part of the amino acid sequences of the Rb proteins among mouse, rat and human. The single-letter amino acid sequence of the mouse, rat and human Rb protein is represented. A vertical bar represents amino acid identity between any two of the three species.
